# Molecular investigation in Chinese patients with primary carnitine deficiency

**DOI:** 10.1002/mgg3.901

**Published:** 2019-07-30

**Authors:** Yanghui Zhang, Haoxian Li, Jing Liu, Huiming Yan, Qin Liu, Xianda Wei, Hui Xi, Zhengjun Jia, Lingqian Wu, Hua Wang

**Affiliations:** ^1^ Hunan Provincial Maternal and Child Health Care Hospital Changsha Hunan China; ^2^ NHC Key Laboratory of Birth Defects Research, Prevention and Treatment (Hunan Provincial Maternal and Child Health Care Hospital) Changsha Hunan China; ^3^ National Research Center for Assisted Reproductive Technology and Reproductive Genetics Jinan Shandong China; ^4^ Center for Medical Genetics, School of Life Sciences Central South University Changsha Hunan China

**Keywords:** newborn screening, primary carnitine deficiency, splice site mutation, variant

## Abstract

**Background:**

Primary carnitine deficiency (PCD) is an autosomal recessive disorder of carnitine transportation caused by mutations in the *SLC22A5* that lead to low serum carnitine levels and decreased intracellular carnitine accumulation. Characteristic clinical findings are hypoketotic hypoglycemia and skeletal and cardiac myopathy.

**Objective:**

To genetically diagnose 24 unrelated Chinese patients with PCD, including 18 infants and six adults.

**Methods:**

The entire coding region and the intron–exon boundaries of *SLC22A5* were amplified by polymerase chain reaction (PCR). In silico analyses and reverse transcription‐polymerase chain reaction (RT‐PCR) were used to predict variants’ impact on protein structure and function.

**Results:**

Disease‐causing variants in the *SLC22A5* were identified in all 24 subjects, and c.288delG, c.495C>A, c.774_775insTCG, c.824+1G>A, and c.1418G>T were novel. The novel variant c.824+1G>A caused a truncated protein p.Phe276Tyrfs*8.

**Conclusions:**

We identified 13 variants in the *SLC22A5* in 24 PCD patients, and five of these variants are novel mutations. c.824+1G>A was confirmed to alter mRNA splicing by reverse transcription PCR. Furthermore, our findings broaden the mutation spectrum of *SLC22A5* and the understanding of the diverse and variable effects of PCD variants.

## 1 INTRODUCTION

Primary carnitine deficiency (OMIM# 212140, PCD) is an autosomal recessive disorder caused by mutations in *SLC22A5* (OMIM# 603377). This gene encodes organic cation transporter type 2 (OCTN2), which transports carnitine across cell membranes. The defective activity of OCTN2 results in urinary carnitine wasting, low plasma carnitine levels, and decreased intracellular carnitine accumulation. Carnitine is necessary for the transfer of long‐chain fatty acids from the cytoplasm into the mitochondria for β‐oxidation (Stanley, 2004). A lack of carnitine results in hypoglycemia by impairing the ability to use fat as an energy source during periods of fasting or stress (Mutlu‐Albayrak et al., 2015). In addition, fat accumulation in the liver, skeletal muscle, and heart leads to hepatic steatosis and myopathy (Magoulas & El‐Hattab, 2012). Furthermore, the clinical manifestations of PCD vary depending on the age of onset and organ involvement, for example, hypoketotic hypoglycemia, hepatomegaly, and hyperammonemia in infants; cardiomyopathy, myopathy, and elevated creatine kinase in childhood; and cardiomyopathy and fatigability in adulthood. In contrast, some individuals with PCD remain asymptomatic throughout their entire life. Plasma‐free carnitine analysis by tandem mass spectrometry can be utilized for PCD screening. Additionally, the measurement of carnitine transport in fibroblasts or genetic testing of the *SLC22A5* could assist in the diagnosis of PCD.

The estimated incidence of PCD is 1:40,000–1:142,000 based on the results of newborn screening (Koizumi et al., 1999; Magoulas & El‐Hattab, 2012; Therrell, Lloyd‐Puryear, Camp, & Mann, 2014; Wilcken, Wiley, Hammond, & Carpenter, 2003). In certain areas, such as the Faroe Islands, PCD is a common disease with an incidence of 1:300 (Rasmussen, Kober, Lund, & Nielsen, 2014; Steuerwald et al., 2017). The incidence of PCD in China is approximately 1:8,938–45,000 among diverse regions (Han et al., 2014, 2012; Ma, 2015; Sun, Wang, & Jiang, 2017); this range is influenced by when and where the epidemiological data were collected. Because of the existence of asymptomatic individuals with PCD, the prevalence of PCD in the general population may be underestimated.

As mentioned above, mutations in the *SLC22A5* cause PCD. The *SLC22A5* spans approximately 30 kb on human chromosome 5q31.1 and comprises 10 exons. Over 150 mutations have been reported in this gene, and most are missense/nonsense. These mutations lead to dysfunctional proteins and disturb carnitine transportation in tissues.

Here, we report 24 unrelated Chinese patients with PCD, including 18 infants who were first suspected via newborn screening and six mothers whose infants failed newborn screening. All subjects had decreased plasma‐free carnitine concentrations and underwent Sanger sequencing of *SLC22A5*, which showed that 22 subjects had compound heterozygous variants, and the remaining two had homozygous variants in *SLC22A5*. Among the variants, five were novel, and c.824+1G>A was confirmed to cause splice site alterations by reverse transcription polymerase chain reaction (RT‐PCR).

## 2 MATERIALS AND METHODS

### 2.1 Patients

Twenty‐four subjects with decreased plasma‐free carnitine levels were initially identified through newborn screening by tandem mass spectrometry in the maternal and child health hospital of Hunan province, China. All infants and their mothers were recalled to repeat plasma carnitine analysis. Eighteen infants sustained low carnitine levels (Table 1), and the remaining six mothers demonstrated low carnitine levels with low or gradually normalized carnitine levels among their babies (Table 2). Other evaluations, including echocardiogram, electrocardiogram, preprandial blood sugar, creatine kinase, and liver transaminase measurements, were conducted for these patients. All infants and mothers were asymptomatic except for an infant with mild mitral and aortic valve regurgitation through echocardiogram and two mothers with easy fatigability. The study was approved by the Ethics Committee of Hunan Provincial Maternal and Child Health Care Hospital.

**Table 1:** Clinical features of 18 infants with primary carnitine deficiency

Subjects	Gender	Plasma‐free carnitine (normal control 10–45 μmol/L)		Clinical presentation
		Initial newborn screening	Recall	
1	F	2.21	1.59	Asymptomatic
2	M	2.95	2.83	Asymptomatic
3	M	1.54	1.41	Asymptomatic
4	M	3.47	4.6	Asymptomatic
5	M	3.8	2.44	Asymptomatic
6	M	5.46	1.41	Asymptomatic
7	M	7.94	4.46	Asymptomatic
8	F	5.28	4.24	Asymptomatic
9	M	5.57	5.57	Asymptomatic
10	F	1.94	1.94	Asymptomatic
11	F	6.16	5.41	Asymptomatic
12	F	7.52	5.85	Mild mitral and aortic valve regurgitation
13	F	6.63	4.8	Asymptomatic
14	F	3.66	4.01	Asymptomatic
15	M	2.35	1.52	Asymptomatic
16	M	2.48	2.52	Asymptomatic
17	F	3.9	2.14	Asymptomatic
18	M	2.46	3.54	Asymptomatic

**Table 2:** Clinical features of six mothers with primary carnitine deficiency

Subjects	Age	Plasma free carnitine (normal control 10–45 μmol/L)			Clinical presentation
		Initial newborn screening of their infants	Recall of their infants	Result of subjects	
19	34 years	2.91	5	2.32	Easy fatigability
20	24 years	3.91	4.78	3.61	Asymptomatic
21	35 years	5.52	9.22	6.82	Asymptomatic
22	31 years	4.17	6.19	3.16	Asymptomatic
23	28 years	2.56	3.65	1.89	Easy fatigability
24	25 years	1.31	8.77	4.75	Asymptomatic

### 2.2 Genetic analysis

The entire coding region and the intron–exon boundaries of *SLC22A5* (NM_003060.3) were amplified by PCR. Dried blood spots were innovatively applied in genetic analysis with 2× T5 Direct PCR Mix blood (TSINGKE Biological Technology, Beijing, China), which was initially designed for the amplification of DNA from whole blood. Primer sequences (Table S1) were designed with PRIMER5 software (PREMIER Biosoft International, Palo Alto, CA, USA). PCR products were sequenced using an ABI PRISM 3100 Genetic Analyzer (Applied Biosystems, Foster City, CA, USA), and sequences were analyzed using DNASTAR (Madison, WI, USA). The genomic sequence of the *SLC22A5* (NM_0030.2) was used as a reference.

RNA was extracted from peripheral blood leukocytes using the TRIzol method. RNA was reverse transcribed into complementary DNA (cDNA) using a RevertAid First Strand cDNA Synthesis Kit (Thermo Scientific, MA, USA). The primers (Table S2) used to amplify the coding regions of *SLC22A5* were designed with PRIMER5. Monoclones were obtained from PCR products with a pClone007 Blunt Simple Vector Kit (TSINGKE Biological Technology, Beijing, China) and DH5α Chemically Competent Cell (TSINGKE Biological Technology, Beijing, China). Monoclones were sequenced using an ABI PRISM 3100 Genetic Analyzer, and the sequences were analyzed using DNASTAR.

## 3 RESULTS

Thirteen different variants were identified in the *SLC22A5* in 24 subjects (Tables 3 and 4). Mutation c.51C>G had the highest frequency of ~ 27% (13/48), followed by c.760C>T and c.1400C>G with frequencies of ~25% (12/48) and ~18.8% (9/48), respectively. Six variants have not been reported in the Human Gene Mutation Database (http://www.hgmd.cf.ac.uk/ac/index.php) or the OCTN2 Database at ARUP Laboratories (http://www.arup.utah.edu/database/OCTN2/OCTN2_display.php). Among these variants, c.288delG and c.824+1G>A were classified as pathogenic; c.495C>A, c.774_775insTCG, and c.1418G>T were classified as likely pathogenic; and c.1298T>C was classified as uncertain significance following the standards and guidelines for the interpretation of sequence variants proposed by the American College of Medical Genetics and Genomics (ACMG) and the Association for Molecular Pathology (AMP) (Richards et al., 2015).

**Table 3:** Variants of SLC22A5 gene in 18 infants with primary carnitine deficiency

Subjects	Variants at nucleotide level	Variants at protein level	References
1	c.495C>A(maternal)	p.Asp165Glu	This study
	c.760C>T (paternal)	p.Arg254*	Tang et al. (2002)
2	c.288delG (paternal)	p.Leu97Trpfs*33	This study
	c.1400C>G (maternal)	p.Ser467Cys	Koizumi et al. (1999)
3	homozygous c.760C>T (maternal/ paternal)	p.Arg254*	
4	c.51C>G(maternal)	p.Phe17Leu	Lee et al. (2010)
	c.338G>A(paternal)	p.Cys113Tyr	Han et al. (2014)
5	c.338G>A(paternal)	p.Cys113Tyr	
	c.760C>T (paternal)	p.Arg254*	
6	c.760C>T (maternal)	p.Arg254*	
	c.1400C>G (paternal)	p.Ser467Cys	
7	c.51C>G (maternal)	p.Phe17Leu	
	c.1298T>C (paternal)	p.Met433Thr	This study
8	c.51C>G (maternal)	p.Phe17Leu	
	c.428C>T (paternal)	p.Pro143Leu	Lee et al. (2010)
9	c.774_775insTCG (paternal)	p.Met258_Leu259insSer	This study
	c.1400C>G (maternal)	p.Ser467Cys	
10	c.51C>G(maternal)	p.Phe17Leu	
	c.760C>T (paternal)	p.Arg254*	
11	c.51C>G (paternal)	p.Phe17Leu	
	c.1400C>G (maternal)	p.Ser467Cys	
12	c.797C>T (maternal)	p.Pro266Leu	Chen et al. (2013)
	c.338G>A(paternal)	p.Cys113Tyr	
13	c.51C>G (paternal)	p.Phe17Leu	
	c.1400C>G (maternal)	p.Ser467Cys	
14	c.51C>G(maternal)	p.Phe17Leu	
	c.1400C>G (paternal)	p.Ser467Cys	
15	c.338G>A (paternal)	p.Cys113Tyr	
	c.760C>T (maternal)	p.Arg254*	
16	c.338G>A (maternal)	p.Cys113Tyr	
	c.824+1G>A (paternal)	p.Phe276Tyrfs*8	This study
17	c.51C>G (paternal)	p.Phe17Leu	
	c.760C>T (maternal)	p.Arg254*	
18	c.51C>G (paternal)	p.Phe17Leu	
	c.760C>T (maternal)	p.Arg254*	

**Table 4:** Variants of SLC22A5 gene in six mothers with primary carnitine deficiency

Subjects	Variants at nucleotide level	Variants at protein level	Variants of infants	References
19	c.760C>T	p.Arg254*	—	
	c.1400C>G	p.Ser467Cys	Heterozygous	
20	c.51C>G	p.Phe17Leu	—	
	c.1400C>G	p.Ser467Cys	Heterozygous	
21	c.760C>T Homozygous	p.Arg254*	Heterozygous	
22	c.51C>G	p.Phe17Leu	—	
	c.1340A>T	p.Tyr447Phe	Heterozygous	Rahbeeni et al. (2002)
23	c.51C>G	p.Phe17Leu	Heterozygous	
	c.1400C>G	p.Ser467Cys	—	
24	c.51C>G	p.Phe17Leu	Heterozygous	
	c.1418G>T	p.Gly473Val	—	This study

**Figure 1:**
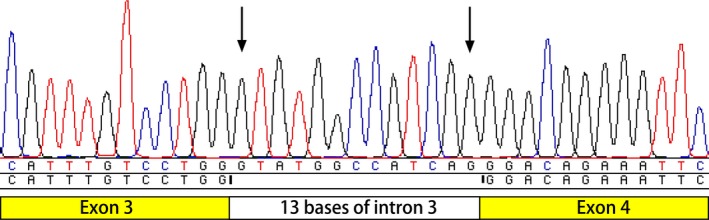
Result of reverse transcription PCR of c.824+1G>A. The variant caused the first 13 bases of intron 3 being included into the coding sequence to form a frameshift of p.Phe276Tyrfs*8

The novel variant c.824+1G>A was confirmed to cause splice site alterations by RT‐PCR and comparison of the sequence with the reference sequence of *SLC22A5* cDNA (NM_0030.2). This variant caused the first 13 bases of intron 3 to be included in the coding sequence, resulting in a truncated protein, p.Phe276Tyrfs*8 (Figure 1).

## 4 DISCUSSION

The broad phenotype of PCD varies from asymptomatic to sudden infant death. A significant number of individuals with PCD are asymptomatic or mildly symptomatic, with symptoms including easy fatigability or decreased stamina (El‐Hattab et al., 2010). However, PCD has risks for significant clinical consequences, such as sudden death triggered by fasting or a catabolic state at any age if left untreated. Early diagnosis and medical interventions are essential for the management of PCD. Carnitine supplementation can efficiently improve symptoms except for those with irreversible consequences, such as central nervous system involvement due to hypoglycemia (Cederbaum et al., 2002). Individuals with PCD exhibit low plasma carnitine levels without carnitine supplementation. There is no clear relationship between plasma carnitine levels and the *SLC22A5* genotype in neonatus ascertained through abnormal newborn screening (Li et al., 2010), which was also observed in our study. No association between genotype and phenotype has been found, particularly among symptomatic patients with identical mutations (Lamhonwah et al., 2002; Longo, Filippo, & Pasquali, 2006). Various mutation types were detected in our study, including missense, nonsense, frameshift, in‐frame insertion, and splice site mutations. All infants were asymptomatic except subject 12 whose echocardiogram indicated mild mitral and aortic valve regurgitation. Two mothers claimed to have easy fatigability, and the remaining four were asymptomatic. The relationship between genotype and phenotype in our study was consistent with previous studies through comparative analyses.

As a maternal disorder can cause secondary carnitine deficiency, plasma carnitine should be evaluated in mothers when the carnitine levels of their infants are low in newborn screening. Using this strategy, a certain number of clinically asymptomatic mothers with mutations were identified (Frigeni et al., 2017; Li et al., 2010). Based on the genetic model of recessive heredity, a certain number of undetected asymptomatic fathers or male adults with PCD are highly probable. Currently, as adult screening might be difficult to implement, examining carnitine levels should be prioritized in individuals who have decreased stamina or easy fatigability.

**Figure 2:**
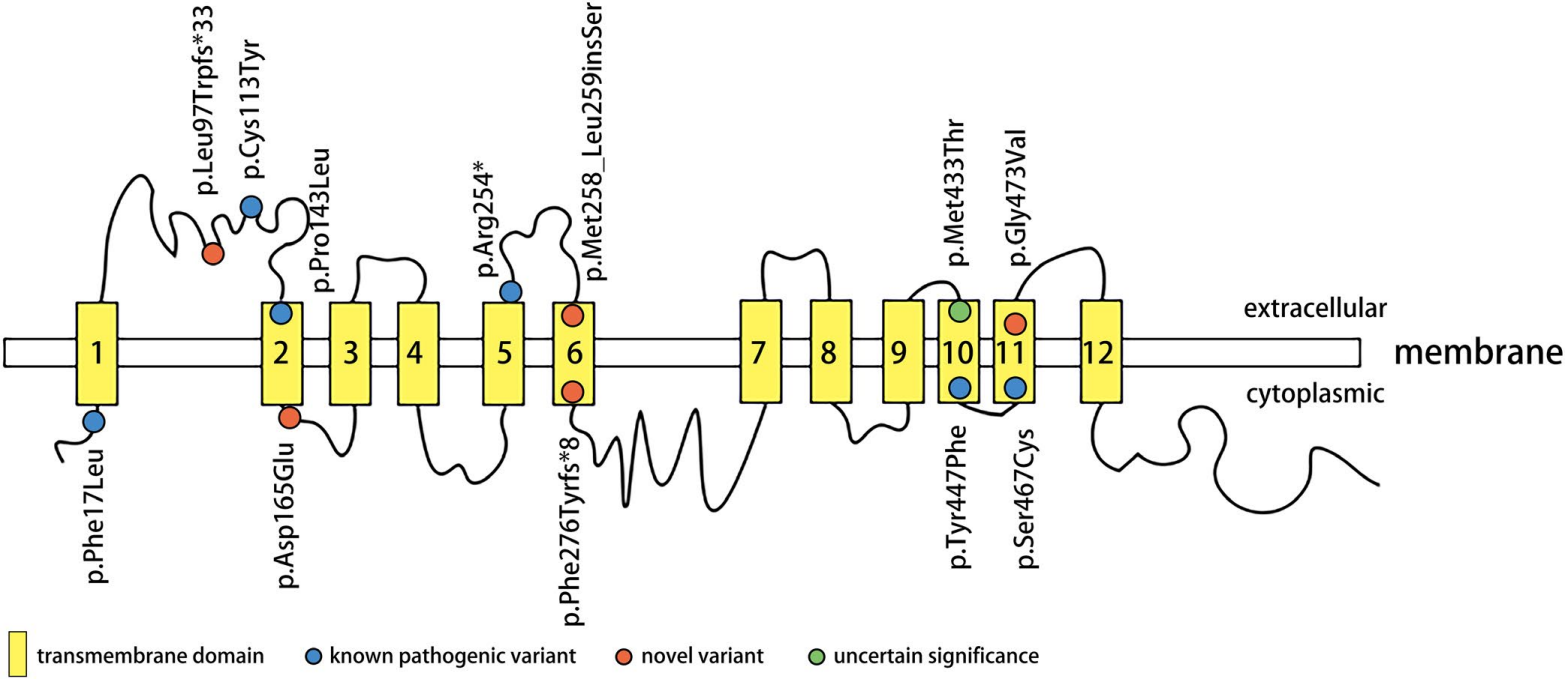
Schematic of the OCTN2 carnitine transporter with location of variants identified in this study. Positions of functional domains are based on the information provided by the Universal Protein Resource (UniProt) (http://www.uniprot.org/)

OCTN2 is a transmembrane protein that transfers carnitine across the cell membrane in a Na^+^‐dependent manner and other organic cations, such as tetraethylammonium (TEA), in a Na^+^‐independent manner. OCTN2 comprises 12 transmembrane domains containing 557 amino acids with both the amino‐ and carboxyl‐terminus in the cytoplasm, similar to other organic cation transporters (Li et al., 2010). The first extracellular loop is highly conserved among organic cation transporters encoded by members of the solute carrier (SLC) 22 family, suggesting an essential role of this loop in transporter function (Burckhardt & Wolff, 2000). Normal glycosylation of the three putative N‐glycosylation sites (Asn‐57, Asn‐64, and Asn‐91) in the first extracellular loop is significant for substrate and sodium recognition (Burckhardt & Wolff, 2000; Wu, Prasad, Leibach, & Ganapathy, 1998). OCTN2 is expressed on the plasma membrane by entering the secretory pathway, including the endoplasmic reticulum and Golgi apparatus (Maekawa et al., 2007). The variant c.824+1G>A causes the first 13 bases of intron 3 to be included in the coding sequence, resulting in a frameshift of p.Phe276Tyrfs*8, a truncation in transmembrane domain 6. Variant c.288delG causes a frameshift of p.Leu97Trpfs*33, which is a termination in the first extracellular loop of OCTN2. These two mutants may lead to a complete absence of the gene product by lack of transcription or nonsense‐mediated decay of an altered transcript. The variant c.774_775insTCG (p.Met258_Leu259insSer) occurs in transmembrane domain 6 and could likely disturb the binding to the cell membrane by inserting a hydroxyl amino acid of serine in a hydrophobic transmembrane domain (Figure 2).

The variants c.495C>A and c.1418G>T are considered pathogenic based on deleterious predictions by the SIFT (http://sift.jcvi.org) and PolyPhen‐2 (http://genetics.bwh.harvard.edu/pph2/) algorithms, while c.1298T>C is predicted to be tolerated by SIFT and pathogenic by PolyPhen‐2. Frigeni et al. (2017) reported that prediction algorithms failed to determine the functional effects of amino acid substitutions in OCTN2 in approximately 20% of cases. Therefore, functional study is the gold standard to confirm the pathogenicity of variants. The measurement of carnitine transport activity in cultured fibroblasts is reliable for diagnosing PCD. Carnitine transport in PCD patients is universally reduced to less than 20% of normal transport in fibroblasts (Longo, Frigeni, & Pasquali, 2016). As this method is time‐consuming and invasive due to the needed skin biopsy, it has not been applied in most medical institutions in China. Moreover, the lack of measurement of carnitine transport activity in cultured fibroblasts is a limitation of this study.

The most frequent mutations of *SLC22A5* have been reported in specific geographical areas, such as c.136C>T in the United States (Frigeni et al., 2017; Li et al., 2010), c.396G>A and c.1400C>G in Japan (Koizumi et al., 1999), and c.95A>G in the Faroe Islands (Rasmussen, Nielsen, et al., 2014). In this study, c.51C>G, c.760C>T, and c.1400C>G were the most frequently occurring mutations, confirming previous findings in the Chinese population (Tables 5). Because of geographical differences and the limited number of test samples, the most frequent mutation is uncertain, but we can safely infer that the three mutations mentioned above are the most frequent mutations of *SLC22A5* in China.

**Table 5:** Frequencies of c.51C>G, c.760C>T, and c.1400C>G of the *SLC22A5* in China

Area	Frequencies			References
	c.1400C>G	c.760C>T	c.51C>G	
Zhejiang	34.3% (23/67)	19.4% (13/67)	11.9 (8/67)	Ma (2015)
Shanghai	2.6% (1/39)	25.6% (10/39)	15.4% (6/39)	Han et al. (2014)
Fujian	31.3 (5/16)	37.5% (6/16)	12.5% (2/16)	Lin et al. (2017)
Jiangsu	50% (7/14)	7.1% (1/14)	14.3% (2/14)	Sun et al. (2017)
Hunan	18.8% (9/48)	25% (12/48)	27.1% (13/48)	This study

All individuals with PCD in this study maintained normal plasma carnitine levels with oral levocarnitine (L‐carnitine) immediately after the initial diagnosis. The infantile metabolic disturbance and childhood myopathy caused by PCD can be fatal without early treatment. Treatment with L‐carnitine supplementation should be initiated early before irreversible organ damage occurs(Magoulas & El‐Hattab, 2012). Antenatal diagnosis has rarely been performed because PCD is treatable, and the treatment method is simple, safe, and effective.

## 5 CONCLUSIONS

In conclusion, we identified 13 variants in the *SLC22A5* in 24 PCD patients, and five of these variants are novel mutations. c.824+1G>A was confirmed to alter mRNA splicing by RT‐PCR. Furthermore, our findings broaden the mutation spectrum of *SLC22A5* and the understanding of the diverse and variable effects of PCD variants.

## CONFLICT OF INTEREST

The authors declare that they have no conflict of interest.

## AUTHOR CONTRIBUTIONS

YZ, LW, and HW chose the topic and designed the experiments; HL, JL, HY, HX, and ZJ collected the patient history and managed the clinical trials; YZ and QL performed the analysis; YZ, HL, and XW analyzed the data; YZ, HL, LW, and HW wrote the manuscript; JL involved in data management and figure modification.
