# Supplementary material for: Molecular investigation in Chinese patients with primary carnitine deficiency
Source: Mol Genet Genomic Med. 2019 Jul 30;7(9):e901. doi: 10.1002/mgg3.901 (PMC6732302; doi:10.1002/mgg3.901)
Supplement: Supplementary file 1 [file MGG3-7-e901-s001.docx]

**Supplementary material**

**Table S1**

Primers and PCR conditions used to amplify the genomic segments of *SLC22A5*.

| **Primer** | **Forward primer (5’–3’)** | **Reverse primer (5’–3’)** | **Annealing temperature (℃)** | **Product size (bp)** |
| --- | --- | --- | --- | --- |
| SLC22A5-1 | CTGGTCGTGCGCCCTATGTA | GGTCTCCATCGCTAGGGTGTT | 58 | 734 |
| SLC22A5-2 | TGTGGGGATGGCAGGATGTT | GGAGGCAAGCCAGGCTACTG | 58 | 498 |
| SLC22A5-3 | CCACTTGGTGGAGCCCATTC | TTCCCTGCCTGTAAGTAAGGTTCA | 58 | 382 |
| SLC22A5-4 | AGGAAGGAACCCAAATTAAACTGC | TTGCTGCCCTCTAGTGAAGGC | 58 | 296 |
| SLC22A5-5 | CTCTTTGCTTCTGGCTTGTGAT | GCTGTAACCTATTCCTCAACTTCTG | 56 | 473 |
| SLC22A5-6 | GACCACCTCTTCTTCCCATACACT | GGTAGGAGACGGGAACTAAATGAC | 58 | 451 |
| SLC22A5-7 | AGGGTTACAGTTACTGCTGCCTTAC | TTTTTCCCACTCCCTCAAAGC | 58 | 555 |
| SLC22A5-8 | CCAGCCTCCTTTCAGCAATC | CAGCTCACATTCAAGCCAGTTAGTA | 58 | 435 |
| SLC22A5-9 | CTTCCAGAGTCCTGGGAGCATA | TGCATCAACACTGACAGAGGAGG | 58 | 257 |
| SLC22A5-10 | CAGTAGCCGCACTGGATAACTCA | GAGGTGCTGAGTCCATCATTGTA | 58 | 509 |

**Table S2**

Primers and PCR conditions used to amplify the cDNA segments of *SLC22A5*.

| **Primer** | **Forward primer (5’–3’)** | **Reverse primer (5’–3’)** | **Annealing temperature (℃)** | **Product size (bp)** |
| --- | --- | --- | --- | --- |
| *SLC22A5*-RT2 | CTTGGAGCTTCGGAGTTCTG | TAAGGAACACATCTGAACGACTTC | 52 | 430 |
